# What happens when you compare yourself to a model eating a cheeseburger? An experiment testing the impact of models promoting calorie-dense foods on beliefs about weight maintenance, body satisfaction, and purchase intent

**DOI:** 10.1186/s40337-020-00335-y

**Published:** 2020-11-04

**Authors:** Kerstin K. Blomquist, Dorothy L. Schmalz, Sarah P. Pate, Alissa Willmerdinger

**Affiliations:** 1 Psychology Department, Furman University, 3300 Poinsett Highway, Greenville, SC 29613 USA; 2 Department of Health, Kinesiology, and Recreation, University of Utah, 70 South 1400 East, 201 Stewart Hall, Salt Lake City, UT 84112 USA

**Keywords:** Food advertisement, Models, Weight maintenance, Body satisfaction

## Abstract

**Background:**

Ads depicting models promoting calorie-dense foods and beverages are ubiquitous and no known research has examined their effects on consumers. Drawing from social comparison theory, we hypothesized that participants who viewed ads with models (versus without models) would be more likely to rate models and less likely to rate themselves as able to consume the calorie-dense foods regularly and still maintain their weight/shape. We also hypothesized that participants who viewed ads with models (versus without models) would report more body dissatisfaction and, consistent with consumer research, a greater intention to purchase the product.

**Methods:**

Participants (*N* = 168) were randomly assigned to view food or beverage ads with models or without models and completed self-report measures.

**Results:**

Participants who viewed ads without models were more likely to rate themselves and most people as able to consume calorie-dense foods regularly and maintain their weight/shape and reported a greater intention to purchase the product. Consistent with our hypotheses, participants who viewed ads with models reported increased body dissatisfaction.

**Conclusions:**

Results indicate that consumers’ perceptions of their own and others’ abilities to regularly consume calorie-dense foods and maintain their weight/shape change based on whether (or not) the calorie-dense foods are promoted by a model. Our findings reveal the nuanced negative effects of calorie-dense food ads with and without models and give insight into the psychological and potential physical health consequences that food ads can have on consumers.

## Plain English summary

Advertisements depicting thin female and muscular male models promoting foods high in calories are ubiquitous, and no known research has examined their effects on consumers. To investigate the impact of models promoting highly caloric foods on consumers, we showed 168 participants a series of food ads either with models or with no models. Then, we asked participants to report their feelings about their body, how likely they could eat the foods advertised and maintain their weight and shape, and how likely they would be to purchase the foods advertised. Results indicated that participants were more intent on buying advertised foods and more likely to believe that the advertised foods would not lead to weight gain when advertised without models. Yet these same products, when advertised with models, were more likely to make consumers feel worse about their bodies. Our findings suggest that food ads with and without models have unique negative effects on consumers.

## Background

Adults are exposed to roughly 362 ads per day [1]. Approximately one quarter of all TV advertisements to adolescents comprise food and beverage ads [2]. Food and beverage companies contribute exorbitant amounts of money on advertisements each year. For example, in 2017, McDonalds spent $1.5 billion on advertising in the United States [3], and Coke spent $899 million [4]. In 2011, children and adolescents were exposed to between 13 and 16 food and beverage ads per day [5], and, in 2015, adults (aged 18–49) viewed just under 7000 food and beverage ads on the television alone [6].

Advertisements frequently include models, celebrities, or athletes (referred to hereafter as models) to promote their products, and the inclusion of models has been shown to be an effective marketing strategy [7]. Provided that the models are attractive, perceived to be trustworthy, and have some level of expertise regarding the product being promoted, consumers respond well to the inclusion of models [8]. Female models most commonly embody an unrealistically thin appearance [9], and male models regularly embody an unrealistically muscular appearance [10, 11]. Research examining the psychological impact of viewing very thin or muscular images indicate that thin ideal images negatively impact female viewers’ body satisfaction [12] while muscular ideal images decrease body satisfaction in men [13, 14].

The bulk of research examining thin or muscular ideal images has involved participants viewing models’ bodies alone or models’ bodies promoting clothes/fashion, cosmetics, and ‘health/fitness.’ Despite the ubiquity of ads promoting calorie-dense foods and sugar-sweetened beverages (referred to hereafter as foods) [15], there is no known research examining the impact of thin or muscular models promoting calorie-dense foods on consumers.

Social comparison theory [16] can help us understand the effect that these ads depicting models promoting calorie-dense foods might have on consumers. Social comparison theory posits that we compare ourselves to others to make better sense of ourselves—of our attributes and abilities [16]. An average person’s appearance comparison to a thin or muscular model would be considered an upward comparison [17]. Research suggests that this comparison results in an acknowledgement of an appearance discrepancy and a negative evaluation of one’s own body shape and weight, resulting in body dissatisfaction [14, 17–19]. Therefore, based on social comparison theory, we would expect that simply viewing the thin or muscular models would result in body dissatisfaction, independent of what the models are promoting.

However, social comparison theory posits that we also compare our *abilities* to others, not just our appearance [16]. Therefore, we posit that viewing models promoting the regular consumption of calorie-dense foods may also lead a person to compare themselves on their ability to regularly consume calorie-dense foods and not gain weight. We propose that when consumers view ads with models promoting calorie-dense foods, the consumers compare themselves to the models’ ability to consume these foods and maintain their weight/shape. We hypothesize that this would also likely be an upward comparison and result in consumers rating themselves as having a poorer ability to regularly consume the calorie-dense foods and maintain their weight/shape.

Understanding the impact that ads depicting models promoting calorie-dense foods has on consumers’ beliefs about weight maintenance is critical when considering the high rates of marketing calorie-dense foods [2, 5, 6], growing rates of obesity [20], and research finding that regular consumption of calorie-dense foods and sugar-sweetened beverages is associated with excess weight gain [21] and type 2 diabetes [22, 23]. Our study addresses an important gap given that these ads have strong potential to negatively impact consumer’s self-evaluations via social comparison and they may have public health implications in their messaging surrounding the consumption of calorie-dense foods and weight management.

This study is a preliminary investigation of the effect of models promoting calorie-dense foods on individuals’ evaluation of weight/shape maintenance ability (WMA), body satisfaction, and food purchase intent. Participants were randomly assigned to view calorie-dense food or beverage ads with or without models. Drawing from social comparison theory, we hypothesized that (1) participants who viewed ads with (versus without) models would rate models as better able to consume the food regularly and maintain their current weight/shape, (2) participants who viewed ads with (versus without) models would to rate themselves as less able to consume the food regularly and still maintain their weight/shape. Furthermore, given the substantial literature highlighting the impact of viewing thin and muscular models on body image [12, 14, 17–19], we hypothesized that (3) participants who viewed ads with (versus without) models would report decreased body satisfaction after viewing the ads. Finally, consistent with consumer research that advertisements depicting models are effective [7, 8], we hypothesized that (4) participants viewing ads with (versus without) models would report greater intent to purchase the food advertised.

## Methods

### Participants

Adults aged 18+ years were recruited to participate in a study “exploring reactions to advertisements” via undergraduate psychology classes at a small southeastern university, messages posted to the college website, Craigslist ads, Google ads, and paper flyers posted around campus and in the surrounding community. Participants received course extra credit or were entered into a raffle for $25. Human subjects approval was obtained by the University’s Internal Review Board. Written informed consent was obtained from all participants.

The final sample (*N* = 168) comprised six community members, 131 introductory psychology, and 31 other undergraduate students. Although the six community participants were slightly older (M = 25 years, *p* < .001), there were no other differences between these participants and university participants on any other demographic, baseline or outcome variable; therefore, we included all participants in the analyses. Demographic and baseline data on our sample are reported in Table 1.

**Table 1:** Differences in demographic, baseline, manipulation check variables between conditions

	**Models (*n*** **= 83)**	**No Models (*n*** **= 85)**			
**Variables**	**Mean ± SD**	**Mean ± SD**	***F***	***p***	***η***_***p***_^***2***^
# Products Recalled	9.1 ± 3.5	9.4 ± 3.2	0.25	.62	.00
Total Media Exposure	6.4 ± 3.7	6.4 ± 3.1	0.01	.94	.00
Age	19.9 ± 3.3	19.8 ± 1.8	0.21	.65	.00
Body Mass Index	22.5 ± 3.0	23.5 ± 5.0	2.65	.11	.02
Pre-Body Satisfaction	5.7 ± 2.5	5.6 ± 2.4	0.06	.80	.00
	**%**	**%**	***X***^***2***^	***p***	
Gender			0.01	.92	
Female	58 (69.9)	60 (70.6)			
Male	25 (30.1)	25 (29.4)			
Race			0.14	.99	
White	70 (84.3)	70 (82.4)			
Black	5 (6.0)	6 (7.1)			
Asian	6 (7.2)	7 (8.2)			
Other, More than one race	2 (2.4)	2 (2.4)			
Ethnicity			0.18	.68	
Hispanic	4 (4.8)	3 (3.5)			
Non-Hispanic	79 (95.2)	82 (96.5)			

### Procedures

Participants were randomly assigned to either food ads with models (*n* = 40), food ads without models (*n* = 43), beverage ads with models (*n* = 43), or beverage ads without models (*n* = 42). Participants first completed pre-assessment scales including the Media Exposure Scale (MES) and Feelings and Attitudes Scale (FAS). Then, participants viewed 20 advertisements. After each ad, participants completed WMA questions and Purchase Intent question. Finally, participants were asked to report the products they remembered as an attention check followed by the FAS again and the Body Image States Scale (BISS). All measures were administered online via SurveyMonkey.

### Measures

#### Media exposure scale (MES)

To gather descriptive data on participants’ frequency of media exposure and to assess whether there were differences between conditions, we administered this 33-item questionnaire [24]. The MES measures frequency and type of media exposure. Participants indicate the amount of time they spend viewing television, magazines, and internet use (e.g., In an average week, how many TOTAL hours do you spend on the Internet?) on a scale from 0 to 50 h/week. Total hours spent on television, magazine reading, and the internet were aggregated into a *Total Media Exposure* score, with higher scores indicating greater media exposure.

#### Feelings and attitudes scale (FAS)

To maintain the cover story while covertly assessing change in body satisfaction pre- and post-viewing ads, participants were asked to indicate their agreement with statements about advertisements as well as questions assessing current satisfaction with physical appearance, skills, personality, and self on a scale from 0 (not at all) to 10 (extremely). The three items specifically measuring body satisfaction were “At this moment, how satisfied are you with your…physical appearance?”, …weight?, and …body shape and size?”. These items were averaged together to create a pre- and post-body satisfaction scale. A body satisfaction change (post-minus-pre) score was used in statistical analyses with negative numbers indicating reduction in body satisfaction from pre-to-post. Cronbach’s alpha was 0.94 at pre-assessment and 0.96 at post-assessment.

#### Weight maintenance ability (WMA) questions

After each advertisement, participants were asked to assess how much they agree that celebrities & models [*Model WMA*], they themselves [*Self WMA*], or people [*People WMA*] in general “could consume the advertised food regularly and still maintain the same weight or body shape without changing current exercise habits” on a scale from 1 (completely disagree) to 11 (completely agree). Higher scores on the WMA items indicate a greater perceived ability to regularly consume the advertised product and maintain weight/shape. Mean Model WMA, Self WMA, and People WMA scores for all 20 ads viewed were computed.

#### Purchase intent

Based on consumer psychology research [25], to assess the impact that the ad conditions might have on purchase intent, participants were asked to indicate how much they intend to purchase the product on a scale from 1 (never) to 11 (definitely). A mean purchase intent score for all 20 ads viewed was computed.

#### Number of products recalled

We asked participants to list which products they remembered seeing advertised to assess attention to the advertisements.

#### Body image states scale (BISS)

The BISS [26] is a valid and reliable, six-item measure of current state body image included to supplement the assessment of pre- and post-body satisfaction. Higher numbers indicate greater body satisfaction; scale ranged from 1 (extremely dissatisfied) to 9 (extremely satisfied). Cronbach’s alpha was 0.84.

#### Demographic

Participants were asked to self-report their age, gender, race/ethnicity, height, and weight after the completion of all other measures. Their reported height and weight were used to calculate body mass index (BMI, kg/m^2^).

### Food and beverage advertisements

We included online picture ads for 20 food and 20 beverage products that were high in calories, sugar, and/or fat such as fast foods, processed convenience foods, sodas, and energy drinks (see Supplemental Table 1). Food and beverage products were selected in ad pairs; only products for which we were able to locate one ad utilizing model endorsement and one that did not were used. Consistent with our anticipated sample gender composition, the ads with models comprised 50% images of men depicting the muscular ideal operationally defined as at least half the man’s body being portrayed with clear muscle definition or half the body of a known celebrity or athlete (e.g., Bradley Cooper and Larry Fitzgerald). The other ads comprised 50% images of women depicting the thin ideal defined as at least half the woman’s body being portrayed with a thin physique (e.g., Beyoncé promoting Pepsi). With regard to racial/ethnic diversity, 40% (8/20) of the ads featured individuals of racial/ethnic minority (see Supplemental Table 1).

As we were specifically interested in understanding the impact of models promoting calorie-dense foods, participants who viewed ads with models were shown both male and female models, independent of their gender, in order to mimic real world marketing of food and beverage ads, which are rarely, if ever, gender-matched to viewers. For this reason, we tested our hypotheses using responses to all 20 advertisements. Furthermore, we did not expect any differences in the impact of foods versus beverages ads on outcome variables a priori and both calorie-dense foods and beverages can negatively impact health. Therefore, for the purpose of testing our hypotheses regarding the effect of using models to promote calorie-dense, low-nutrient products, we combined food and beverage ad conditions and compared participants who viewed either food or beverage ads with models to those who viewed either food or beverage ads without models. There were no baseline or demographic differences between participants in food versus beverage conditions.

### Statistical analyses

Analyses were conducted using SPSS version 25. To test for demographic, baseline or attention check differences across conditions, we conducted Analyses of Variance (ANOVAs) with continuous variables and chi-square analyses with categorical variables. Pearson Bivariate correlations examined associations among variables. To test our primary hypotheses regarding the impact of viewing ads with versus without models on WMA ability, body satisfaction, and purchase intent, we conducted a series of ANOVAs. We report effect sizes with partial eta squared where a value of .01 is considered small, .06 is considered medium, and .14 is considered large [27].

Given that the average effect size for the impact of thin images on women’s body satisfaction in experimental and correlational studies is small-to-moderate [12], we estimated our required sample size to test our primary hypotheses using G*Power 3.1 with a small-to-moderate effect size (*f* = .20) with power at .8, which indicated that the total sample size needed to conduct an ANOVA with 2 groups (with vs without models) was *N* = 200. A post-hoc analysis indicated the power achieved with our sample of *N* = 168 with small-to-moderate effect sizes was .73.

## Results

There were no significant differences between conditions on any demographic, baseline, and attention check variable (see Table 1). On average, participants recalled 9/20 products and took 33.3 ± 19.0 min to complete the entire survey, suggesting that participants paid sufficient attention to completing the measures and viewing the advertisements. The majority of participants were female (70%), White (83%), non-Hispanic (96%) and within the normal range for BMI (80%, 18.5–24.9 kg/m^2^).

### Correlations among study variables

Significant correlations indicate that the more satisfied participants were with their appearance, the more they perceived models and they themselves as capable of regularly consuming calorie-dense foods and not gaining weight (see Table 2). Similarly, participants with a lower BMI reported greater body satisfaction and were more likely to believe that they themselves could consume the calorie-dense foods regularly and still maintain their weight/shape. The more participants perceived models, themselves, and other people as capable of regularly consuming calorie-dense foods and not gaining weight, the greater their intent to purchase the advertised product.

**Table 2:** Pearson bivariate correlations among variables

	(1)	(2)	(3)	(4)	(5)	(6)
1. BMI						
2. Model WMA	−.04					
3. Self WMA	−.17*	.83**				
4. People WMA	−.05	.93**	.86**			
5. Body Satisfaction Change	−.06	.09	.15	.12		
6. BISS	−.30*	.15*	.33**	.12	.12	
7. Purchase Intent	−.04	.24**	.29**	.27**	.01	.06

*BMI* Body mass index, *Model WMA* Celebrity, model, or athlete in the ad can consume the advertised product regularly and still maintain their weight/shape without changing exercise, *Self WMA* Participant can consume the advertised product regularly and still maintain their weight/shape without changing exercise, *People WMA* Most people can consume the advertised product regularly and still maintain their weight/shape without changing exercise, *Body Satisfaction Change* (post-minus-pre), *BISS* Body Image States Scale

*p < .05, **p < .01

### Ads with models versus no models

Table 3 presents analyses comparing participants who viewed ads with versus without models. Contrary to our hypothesis (1), there was no difference between participants who viewed ads with (versus without) models on ratings of models’ ability to regularly consume the calorie-dense foods and maintain weight/shape. Consistent with our hypothesis (2), participants who viewed ads with (versus without) models rated themselves as less able to consume the calorie-dense foods and still maintain their weight/shape without changing their exercise habits. Participants who viewed the ads with (versus without) models also rated other people as less able to consume the calorie-dense foods and still maintain their weight/shape without changing their exercise habits. Consistent with our hypothesis (3), body satisfaction decreased from pre-to-post for participants who viewed ads with models and slightly increased for participants who viewed ads without models. Contrary to our hypothesis (4), participants who viewed ads with (versus without) models reported being less likely to purchase the product.

**Table 3:** Comparison of ads with models versus no models

	Models (***n*** = 83)	No Models (***n*** = 85)			
Variables	Mean ± SD	Mean ± SD	***F***	***p***	***η***_***p***_^***2***^
Model WMA	3.4 ± 1.9	3.5 ± 1.8	0.03	.87	.00
Self WMA	3.1 ± 2.1	3.8 ± 2.3	3.87	.05	.02
People WMA	3.0 ± 1.6	3.6 ± 1.7	5.08	.03	.03
Body Satisfaction Change	−0.1 ± 0.9	0.2 ± 0.7	4.49	.04	.03
BISS	5.5 ± 1.7	5.5 ± 1.3	0.01	.92	.00
Purchase Intent	3.0 ± 1.2	3.8 ± 1.7	11.25	.00	.06

*Model WMA* Celebrity, model, or athlete can consume the advertised product regularly and still maintain their weight/shape without changing exercise, *Self WMA* Participant can consume the advertised product regularly and still maintain their weight/shape without changing exercise, *People WMA* Most people can consume the advertised product regularly and still maintain their weight/shape without changing exercise, *Body Satisfaction Change* (post-minus-pre) are reported in this table, *BISS* Body Image States Scale

### Exploratory analyses

Although the goal of the study was to examine the effect of using models to promote calorie-dense foods, since participants viewed ads with both male and female models and provided WMA ratings after each ad, we explored whether results differed by the gender of the model in ads. Table 4 presents the results from these exploratory paired samples t-tests. Results indicate that both female and male participants rated models, themselves, and others as less able to consume the calorie-dense foods and still maintain their weight/shape without changing their exercise habits when viewing female models compared to viewing ads with male models.

**Table 4:** Paired sample t-tests comparing impact of viewing female models vs male models on beliefs about weight maintenance

	Female Models	Male Models			
	Mean ± SD	Mean ± SD	***t***	***p***	***d***
**Women (*n*** **= 58)**					
Model WMA	2.6 ± 1.7	3.7 ± 2.0	-7.77	.00	−.61
Self WMA	2.4 ± 1.9	2.8 ± 1.9	-3.79	.00	−.20
People WMA	2.3 ± 1.4	3.3 ± 1.9	-7.44	.00	−.62
**Men (*n*** **= 25)**					
Model WMA	3.2 ± 1.6	4.9 ± 2.3	-6.36	.00	−.84
Self WMA	3.7 ± 2.0	4.6 ± 2.3	-3.37	.00	−.40
People WMA	2.8 ± 1.4	4.0 ± 2.0	-6.37	.00	−.72

*Model WMA* Celebrity, model, or athlete can consume the advertised product regularly and still maintain their weight/shape without changing exercise, *Self WMA* Participant can consume the advertised product regularly and still maintain their weight/shape without changing exercise, *People WMA* Most people can consume the advertised product regularly and still maintain their weight/shape without changing exercise

## Discussion

The primary goal of this study was to examine the effect of models promoting calorie-dense foods and beverages on perceptions of weight/shape maintenance ability, body satisfaction, and food purchase intent. Results provide preliminary support for two of our four hypotheses with small-to-medium sized effects. Participants viewing ads with (versus without) models as a point of comparison perceived themselves as less able to regularly consume the advertised products and maintain their weight/shape and reported a decrease in body satisfaction after viewing the ads, consistent with previous body image research [12]. After viewing ads with (versus without) models, participants were also more likely to rate other people as less able to regularly consume the advertised products and maintain their weight/shape and reported less intention to purchase the product. Altogether, our findings suggest that the use of models to promote calorie-dense foods has mixed implications.

In accordance with social comparison theory, participants appear to compare themselves (whether consciously or unconsciously) to the models, athletes, or celebrities endorsing the products. Our findings suggest that the image of the model seems to serve as a point of comparison for participants. Consistent with social comparison theory, we proposed that participants would compare their *ability* to consume the food regularly and maintain their weight/shape with the model’s *ability* to regularly consume the product and maintain their weight/shape. However, it is also possible that participants were not comparing themselves to the model’s *ability* to consume the food regularly but rather to the model’s weight/shape. That is, it could be that the consumers were evaluating their ability to achieve the thin or muscular ideal presented by the model in the ad, leading them to conclude that they would not be able to achieve that level of thinness or muscularity if they were to regularly consume the food promoted.

It is also possible that participants simply do not believe that models regularly consume the calorie-dense foods that they are promoting. This may be more true of female versus male models because, for women, thinness is often perceived as being achieved by decreasing caloric intake or dietary restriction, whereas for men, muscularity is perceived as being achieved by increasing caloric intake [28]. Our finding that participants were more likely to believe models, they themselves, and other people could regularly consume the calorie-dense food and maintain their weight/shape when the food was promoted by male (versus female) models supports the idea that consumers may not believe that female models actually consume the food they are promoting, but that male models do. More research is needed to explore what comparisons participants are specifically engaging in when viewing female versus male models promoting calorie-dense foods. Furthermore, our findings suggest that the impact on female participants might be more pronounced if they had only viewed ads with female models. Future research should test the impact of gender-matched models for both women and men.

Contrary to our hypothesis, there was no difference in ratings of models’ WMA between participants who viewed ads with versus without models. It is possible that consumers can easily imagine idealized thin/muscular models regardless of whether the model is directly presented to them. Consumers may have internalized images of male and female models that are easily accessible for comparison purposes.

Interestingly, participants not only rated their own weight maintenance ability but most people’s weight maintenance ability as lower when viewing ads with (versus without) models. This suggests that models may have served as a point of comparison when evaluating other people’s weight maintenance ability. It is possible that ads portraying models promoting calorie-dense foods could set a comparison standard by which consumers judge others about their weight/shape and might contribute to weight stigma in our society [29].

Consistent with body image research [12, 14, 17–19], participants who viewed ads with (versus without) models reported a small but significant decrease in body satisfaction after viewing the ads. However, we did not find the lower body satisfaction scores among participants who viewed ads with (versus without) models on the Body Image States Scale (BISS). It is possible that the Feelings and Attitudes (FAS) body satisfaction measure assesses a slightly different aspect of body satisfaction than the BISS does or that the BISS is less sensitive of a measure. Although there were no baseline differences on the FAS measure of body satisfaction between participants who viewed ads with (versus without) models, we did not administer the BISS at baseline so cannot determine whether or not there was a difference in baseline BISS scores or a difference in change in BISS scores from before to after viewing the ads. In addition, it is possible that the effect on the first scale is a spurious finding or that the observed decrease in body satisfaction is more a reaction to having had to answer questions about weight maintenance abilities and intent to purchase the calorie-dense food products after every ad viewed. However, given that participants who viewed ads without models completed the same questions, the observed decrease in body satisfaction on one scale can likely be attributed to the difference in experimental conditions—viewing ads with models versus without models.

Contrary to consumer research that ads depicting models is an effective marketing strategy, we found that participants reported a lower intent to purchase foods after viewing advertisements with models. It is possible that engaging in social comparison with a thin/muscular model and reflecting on weight maintenance while experiencing lower body satisfaction led participants to be less likely to purchase the foods advertised. This is consistent with recent research that found viewing thin ideal images led to decreased food consumption [30].

To our knowledge, no one has investigated the negative impact of model endorsement of calorie-dense foods despite their ubiquity, their potential to convey inaccurate messages regarding consumption of these foods and weight maintenance, and their negative impact on consumer’s self-evaluations. We tested our hypotheses in an experiment using random assignment, an attention check, and validated scales where available. Our study also had several limitations. Even after collapsing food and beverage ad conditions, our study was slightly underpowered, which may explain the marginally significant finding for self WMA (*p* = .05). Our sample did not include enough men to test our hypotheses separately by gender. Given that research has found that women are especially apt to engage in appearance comparison with images in the media [18, 31] and that women report overall greater weight/shape concerns relative to men [32], future research should explore the impact of food ads with models separately by gender in a larger sample of men and women. Our exploratory analyses on gender differences and exposure to female versus male models yielded some compelling outcomes that warrant further investigation, but we are wary of drawing any major conclusions because of limitations in sample size and composition. Our study design is limited in only exploring the impact of models promoting calorie-dense foods and beverages versus low calorie or no calorie foods. Future research should consider testing the impact of these ads using a larger, more diverse sample, controlling the exact amount of time participants viewed each ad, including additional control groups (e.g., ads with and without models for low/no calorie foods), examining potential moderators (e.g., age, gender, gender-matched vs cross-gender models, history of dieting or binge eating), and including a measure of eating psychopathology as well as behavioral outcome variables (e.g., food consumption).

## Conclusions

Our findings present an interesting paradox. When advertised without a model, calorie-dense foods and beverages are more likely to be purchased and consumers are less likely to believe that their regular consumption would lead to weight gain, yet these same products advertised with models are more likely to decrease body satisfaction, which is associated with a host of psychological and physical problems (e.g., disordered eating, depression, stress, etc.) [33, 34]. It appears that fewer food/beverage ads may be the most helpful, especially for those most vulnerable to influences from food ads (e.g., children and adolescents) [35].

## Supplementary information

**Additional file 1: Supplemental Table 1.** Food and Beverage Products Featured in Advertisements in randomized order of presentation.

## Data Availability

The datasets used and analyzed during the current study are available from the corresponding author on reasonable request.

## Abbreviations

BMI: Body mass index
WMA: Weight maintenance ability
BISS: Body image states scale
