# Supplementary material for: What happens when you compare yourself to a model eating a cheeseburger? An experiment testing the impact of models promoting calorie-dense foods on beliefs about weight maintenance, body satisfaction, and purchase intent
Source: J Eat Disord. 2020 Nov 4;8:55. doi: 10.1186/s40337-020-00335-y (PMC7640395; doi:10.1186/s40337-020-00335-y)
Supplement: Supplementary file 1 — Additional file 1: Supplemental Table 1. Food and Beverage Products Featured in Advertisements in randomized order of presentation. [file 40337_2020_335_MOESM1_ESM.docx]

**Supplemental Table 1. Food and Beverage Products Featured in Advertisements in randomized order of presentation**

|  | **Food Products** | **Model Gender** | **Race/Ethnicity** |
| --- | --- | --- | --- |
| **1** | Kit Kat | Female | White |
| **2** | Hardees | Female | Non-White |
| **3** | Dunkin Donuts | Female | White |
| **4** | Dove Chocolate | Male | White |
| **5** | Doritos | Male | Non-White |
| **6** | Godiva | Female | White |
| **7** | Oreo | Male | White |
| **8** | Taco Bell | Female | White |
| **9** | M&Ms | Female | White |
| **10** | KFC | Male | White |
| **11** | Hooters | Female | Non-White |
| **12** | Haagen Daaz | Male | White |
| **13** | Snickers | Female | Non-White |
| **14** | IHOP | Male | Non-White |
| **15** | Twizzlers | Male | White |
| **16** | McDonalds | Male | Non-White |
| **17** | Duncan Hines | Female | Non-White |
| **18** | Butterfingers | Male | Non-White |
| **19** | Pizza Hut | Male | White |
| **20** | Lays | Female | White |
|  | **Beverage Products** |  |  |
| **1** | Snapple | Male | Non-White |
| **2** | Diet Dr. Pepper | Male | White |
| **3** | Fanta | Female | Non-White |
| **4** | Crystal Light | Female | Non-White |
| **5** | Powerade | Male | Non-White |
| **6** | Starbucks | Male | White |
| **7** | RockStar | Female | White |
| **8** | Sprite | Male | White |
| **9** | Lipton Tea | Male | White |
| **10** | Pepsi | Female | Non-White |
| **11** | 7UP | Female | White |
| **12** | Vault | Male | White |
| **13** | Burn | Female | White |
| **14** | AMP | Male | White |
| **15** | Fuze | Male | Non-White |
| **16** | Gatorade | Female | Non-White |
| **17** | Monster | Female | White |
| **18** | Sierra Mist | Female | White |
| **19** | Vitamin Water | Male | Non-White |
| **20** | Cheerwine | Female | White |

**Note.** The beverage products comprised sugar-sweetened beverages with two overlooked exceptions: Diet Dr. Pepper and Crystal Light. Diet Dr. Pepper ad ratings on Model WM, Self WM, People WM, and Purchase Intent did not significantly differ from the mean ratings for the other 18 beverage ads (*p* > .05). We therefore retained Diet Dr. Pepper scores in all analyses. On the other hand, Crystal Light ad ratings on Model WM, Self WM, and People WM were significantly different from the mean scores for the other 18 ads (*p*<.001, *p*<.001, *p*<.001, *p*=.007, respectively). Although ratings on Purchase Intent was not significantly different from the mean scores for the other 18 ads, we removed the Crystal Light scores for Model WM, Self WM, People WM from the analyses.

For the food ads, three ads featured African-American men, two ads featured African-American women, two ads featured Latino men, and one ad featured a Latina woman. For the beverage ads, three ads featured African-American men, three ads featured African-American women, and two ads featured Hispanic men.
